# Rebaudioside B Attenuates Lung Ischemia-reperfusion Injury Associated Apoptosis and Inflammation

**DOI:** 10.2174/0127722708295154240327035857

**Published:** 2024-04-05

**Authors:** Xiangyang Wu, Tao Qiao, Jian Huang, Jian Li, Shilin Wei, Jianbao Yang, Yanchun Zhang, Yongnan Li

**Affiliations:** 1 Department of Cardiac Surgery, Lanzhou University Second Hospital, Lanzhou University, Lanzhou, China;; 2 Department of Thoracic Surgery, Division of Life Sciences and Medicine, The First Affiliated Hospital of USTC,University of Science and Technology of China, Hefei, China;; 3 Department of General Surgery, Lanzhou University Second Hospital, Lanzhou University, Lanzhou, China;; 4 Department of Thoracic Surgery, Lanzhou University Second Hospital, Lanzhou University, Lanzhou, China

**Keywords:** Rebaudioside B, lung ischemia-reperfusion injury, apoptosis, high-throughput screening, oxygen-glucose deprivation/recovery, A549

## Abstract

***Objective*:** At present, no proven effective treatment is available for Lung Ischemia-reperfusion Injury (LIRI). Natural compounds offer promising prospects for developing new drugs to address various diseases. This study sought to explore the potential of Rebaudioside B (Reb B) as a treatment compound for LIRI, both *in vivo* and *in vitro*.

***Methods*:** This study involved utilizing the human pulmonary alveolar cell line A549, consisting of epithelial type II cells, subjected to Oxygen-glucose Deprivation/recovery (OGD/R) for high-throughput *in vitro* cell viability screening. The aim was to identify the most promising candidate compounds. Additionally, an *in vivo* rat model of lung ischemia-reperfusion was employed to evaluate the potential protective effects of Reb B.

***Results*:** Through high-throughput screening, Reb B emerged as the most promising natural compound among those tested. In the A549 OGD/R models, Reb B exhibited a capacity to enhance cell viability by mitigating apoptosis. In the *in vivo* LIRI model, pre-treatment with Reb B notably decreased apoptotic cells, perivascular edema, and neutrophil infiltration within lung tissues. Furthermore, Reb B demonstrated its ability to attenuate lung inflammation associated with LIRI primarily by elevating IL-10 levels while reducing levels of IL-6, IL-8, and TNF-α.

***Conclusion*:** The comprehensive outcomes strongly suggest Reb B's potential as a protective agent against LIRI. This effect is attributed to its inhibition of the mitochondrial apoptotic pathway and its ability to mitigate the inflammatory response.

## 1 INTRODUCTION

Lung Ischemia-reperfusion Injury (LIRI) manifests as a complex pathophysiological process observed across various clinical scenarios, such as cardiac arrest, trauma, pulmonary thrombosis, lung transplantation, and cardiopulmonary bypass [1]. Pulmonary ischemia-reperfusion injury encompasses damage to both the pulmonary vascular endothelium and alveolar epithelium [2]. LIRI stands as the primary cause of early primary graft dysfunction and subsequent failure following lung transplantation [3]. In this context, natural compounds may represent a promising source of new compounds to treat LIRI. Natural products constitute a vast reservoir of diverse chemical compounds characterized by unique biological targets and mechanisms of action [4]. In this study, a high-throughput screening of a compound library consisting of 2,661 individual compounds derived from natural sources was performed. The aim was to identify the most potent compound using an *in vitro* model of ischemia-reperfusion injury. The study utilized A549 cells, a human pulmonary alveolar cell line exhibiting properties akin to epithelial type II cells. The findings revealed Rebaudioside B (Reb B) to be the most promising compound. Reb B is a minor component of Steviol Glycosides (SGs) that can be found mainly in stevia leaf [5]. The SGs despite being 300 times sweeter than sucrose are non-caloric and, therefore, do not increase blood sugar levels [6]. For the above-mentioned reasons, SGs are widely used as a non-toxic food additive for sweeteners [7]. In this context, Reb B was chosen for this study due to its substantial inhibition of apoptosis. The current study aimed to explore the potential protective effects of Reb B on an *in vivo* model of LIRI.

## 2 MATERIALS AND METHODS

### 2.1 Potential Target Proteins of Reb B and LIRI

The potential targets of Reb B in Homo sapiens were explored using online databases, such as PubChem (https://pubchem.ncbi.nlm.nih.gov/) and Pharmmapper (http://www.lilab-ecust.cn/pharmmapper/). Additionally, known targets from prior studies were acquired from the PubMed database (http://pubmed.cn/). These targets were amalgamated with those from the aforementioned sources and then refined to eliminate redundancy, forming the comprehensive pool of Reb B-related targets.

Moreover, disease target data associated with LIRI were retrieved from online databases, such as Online Mendelian Inheritance in Man (https://www.ncbi.nlm.nih.gov/omim) and Genecards (https://www.genecards.org/). After consolidating these targets, redundancies were eliminated, establishing them as the relevant targets of LIRI.

Utilizing the UniProt database (https://www.uniprot.org/), target screening was conducted, restricting the targets to the species 'Homo sapiens,' ensuring standardization of the target genes.

### 2.2 GO Enrichment Analysis

Enrichment analysis for GO biological processes was conducted using the DAVID 6.8 database (https://david.ncifcrf.gov/). The GO enrichment analysis encompassed three dimensions: molecular function, biological process, and cellular component. The enrichment criteria included a *p*-value cutoff of 0.05 and a Q-value cutoff of 0.05, with all other settings left at their default values. The top 10 enriched terms in each category were selected, and the results were subjected to visual analysis using bioinformatics tools (http://www.bioinformatics.com.com.cn/) based on the *p*-values, Q-values, and the count of genes enriched in each term.

### 2.3 Ethical Statement

The animal experimental protocols for this study received approval from the Institutional Animal Experimental Ethics Committee of Lanzhou University Second Hospital (code: D2077-026; location: Lanzhou, Gansu, China). All animal-related experimental procedures strictly adhered to the Animal Care Guidelines set by the National Institutes of Health and complied with the Animal Research: Reporting of *In vivo* Experiments (ARRIVE) guidelines. Hence, every possible effort was made to alleviate the animals' suffering and minimize the overall number of animals used in the study. A549 cells were treated according to the approved protocol by the Ethics Committee of Lanzhou University Second Hospital (2022B-343) and strictly adhered to the guidelines of the Helsinki Declaration.

We purchased 30 male Sprague Dawley rats, aged 8 weeks and weighing approximately 250±25 grams, from the Lanzhou Veterinary Research Institute in Lanzhou City for the experiment. The present study comprised the *in vitro* (Experiment 1) and the *in vivo* (Experiment 2) studies to assess the efficacy of Reb B in LIRI. Experiment 1 was designed to screen the most promising compound from natural product libraries (2,661 compounds) that could inhibit ischemia-reperfusion injury of A549 cells. Experiment 2 investigated the impact of Reb B on lung function and inflammation following ischemia-reperfusion injury in rats.

## 3 EXPERIMENT 1

### 3.1 Establishing the OGD/R Model Within the A549 Cellular Environment

The A549 cell line was sourced from the Cell Bank of the Chinese Academy of Sciences (Shanghai, China). These cells were maintained in Dulbecco's Modified Eagle Medium (DMEM) (Solarbio, Beijing, China) supplemented with 10% fetal calf serum (FBS) (GIBCO, USA) and 100U/ml penicillin and 200 μg/ml streptomycin (GIBCO, USA). The cells were cultured in a humidified incubator (Thermo Fisher, Waltham, USA) at 37°C with 5% CO_2_. The culture medium was refreshed every 48 hours. For *in vitro* simulation of LIRI, A549 cells underwent OGD/R. This involved subjecting cells to sugar-free and serum-free DMEM medium and incubating them in a tri-gas incubator (37°C, 1% O_2_, 5% CO_2_) (Thermo Fisher, Waltham, USA). After 12 hours of OGD exposure, the fresh culture medium was replaced, returning the cells to normal culture conditions.

### 3.2 Compound Candidates Identified Through High-throughput Screening and Evaluation of the Effect on A549 Cells OGD/R Model

A549 cells were seeded onto 96-well plates and allowed to adhere for 24 hours before initiating subsequent procedures. Before OGD/R, cells were pretreated with each of 2,661 natural compounds from a natural product library at a final concentration of 10 µM for 12 h. Following OGD/R treatment, the CCK8 cell proliferation kit (Biosharp, Hefei, China) was employed following the manufacturer’s guidelines. Quantitative analysis of cell viability allowed us to identify the most promising compound, Reb B. To study the cytotoxic effects of Reb B on A549 cells under the OGD/R model, different concentrations were tested, namely 1 mM, 10 mM, 100 mM, and 1000 mM. To understand the role of the most promising candidates in the A549 cells OGD/R model, three groups of cells were used, namely the vehicle group, the OGD/R group, and the OGD/R+compound candidates group. The cells before OGD/R modeling were incubated with a concentration of 10mM Reb B. The protective mechanism of Reb B was studied by Hoechst 33258 staining, Reverse Transcription-quantitative PCR (RT-qPCR), and Western Blot (WB). All experiments underwent triplicate trials and were repeated a minimum of three times for validation.

## 4 EXPERIMENT 2

### 4.1 Animals

Thirty male Sprague Dawley rats, 8 weeks old and weighing approximately 250±25 g, were utilized in this investigation. The animals were kept in plastic cages with smooth flooring, maintained at a temperature of 22°C under a 12-hour light/dark cycle. All experiments were carried out during the light phase of this cycle, precisely from 8:00 am to 6:00 pm.

## 5 EXPERIMENTAL LIRI *IN VIVO* MODEL

The rats were randomly allocated to three distinct groups: the Sham group (n=5), the LIRI group (n=6), and the LIRI+Reb B group (n=6). Reb B was intravenously injected 60 min before thoracotomy as a pretreatment. The animal models were anesthetized by administering 2% sevoflurane after an intraperitoneal injection of 10% chloral hydrate at a dosage of 0.3 ml/kg for sedation. They were then positioned in a supine orientation to ensure immobilization. After anterior neck soft tissue dissection and sterilization to expose the trachea, tracheotomy was performed, and artificial ventilation was started (tidal volume 8 ml/kg, frequency 72/min, positive end-expiratory pressure of 2 cm H_2_O, FiO_2_ 100%). Reb B (5 mg/kg) dissolved in Dimethylsulfoxide (DMSO) was administered through the femoral vein in the LIRI+Reb B group, and an equal volume of DMSO was administered to both the Sham and LIRI groups. The animals were positioned in the left lateral decubitus posture, followed by a left thoracotomy executed through the fourth or fifth intercostal space. Careful dissection of the muscular layer and pleura allowed for full exposure of the heart and lungs. In the LIRI groups treated with vehicle or Reb B (LIRI and LIRI+Reb B groups, respectively), ischemia was induced by non-invasively occluding the left pulmonary hilum using a vascular clamp for 90 minutes. During the left lung ischemia, tidal volume parameters dropped to 5 ml/kg. Following the 90-minute ischemic period, the non-invasive vascular clamp was removed to initiate a 120-minute reperfusion phase, during which the tidal volume was restored to 8mL/kg. The rats in the Sham group underwent uninterrupted perfusion for 210 minutes without experiencing ischemia. After the experiments, the lung tissues and BALF were collected for the following examination.

### 5.1 Chemicals and Antibodies

The natural product library utilized in this study was acquired from Selleck Chemicals (#L1400, Houston, USA), comprising a collection of 2,661 natural compounds (≥99% purity) provided as solutions dissolved in DMSO and water. Primary antibodies utilized encompassed anti-Bcl2, anti-Bax, and anti-caspase-3 obtained from Proteintech (Wuhan, China), anti-cleaved caspase-3 sourced from Cell Signaling Technology (Danvers, MA, USA), and anti-myeloperoxidase procured from Cambridge, MA, USA.

### 5.2 The Cell Counting Kit-8 (CCK-8) Assay

Cell viability was evaluated using a Cell Counting Kit-8 (CCK-8, Biosharp, Hefei, China) following the guidelines provided by the manufacturer. The absorbance (A) was measured at a wavelength of 450 nm using a microplate reader (Thermo Fisher, Waltham, USA). The formula used to ascertain cell survival rate is as follows: Cell viability (%) = (A__experiment_ – A__blank_) / (A__control_ - A__blank_) × 100%.

### 5.3 Hoechst 33258 Staining

Morphological analysis was conducted *via* Hoechst 33258 staining, aiming to investigate cell apoptosis. After treatment, cells were washed with Phosphate-buffered Saline (PBS) and subsequently fixed in 4% paraformaldehyde at room temperature for 15 minutes. Subsequently, the cells underwent a 10-minute incubation period with Hoechst 33258 staining at room temperature, after which observation was carried out utilizing a fluorescence microscope (Olympus, Tokyo, Japan). Apoptotic cells exhibited intense fluorescence within their nuclei, whereas non-apoptotic cells displayed comparatively weaker fluorescence levels. The apoptotic rate was determined by tallying the count of apoptotic cells within 100 cells under a randomly selected field of view, employing the formula: Apoptosis cell rate = (number of apoptotic cells / total number of cells) × 100%.

### 5.4 The Western Blot Analysis

The entirety of protein content extracted from the cells and lower left lung tissues was extracted using Radioimmunoprecipitation Assay buffer (RIPA buffer), lysis buffer (50mM Tris (pH 7.4), 1% Triton X-100, 1% sodium deoxycholate, 150mM NaCl, 0.1% SDS, and sodium orthovanadate with protease inhibitor. The supernatants were gathered and evaluated for protein concentration through the utilization of the Bicinchoninic Acid (BCA) assay. The Sodium Dodecyl Sulfate-polyacrylamide Gel Electrophoresis (SDS-PAGE) technique was employed to transfer the proteins onto 0.45μm Polyvinylidene Fluoride (PVDF) membranes. Before overnight incubation at 4°C with primary antibodies, the membranes were blocked with 5% defatted milk for 2 hours at room temperature. In this experiment, β-actin (Proteintech, Wuhan, China) served as the reference gene. Finally, positive signals were developed by SuperSignal West Pico Plus ECL Kit (Thermo Fisher, Waltham, USA) and analyzed using ImageJ 1.49 (National Institutes of Health, USA).

### 5.5 Reverse-transcription Quantitative PCR (RT-qPCR) Analysis

RNA extraction from both cells and lung tissues in each group was carried out using Trizol reagent (Takara, Kusatsu, Japan) following the manufacturer’s protocol. RNA yield was evaluated using a NanoDrop spectrophotometer (Thermo Fisher, Waltham, USA), and 1000ng of RNA was utilized for complementary DNA synthesis, followed by PCR amplification. In this study, real-time PCR was performed using TB Green qPCR Mix Plus (Takara, Kusatsu, Japan) and the CFX96TM Real-time Detection System (Bio-Rad, CA, USA), with β-actin serving as the endogenous reference. The data underwent analysis following the manufacturer's protocol, employing the relative standard curve method. All data were normalized relative to β-actin mRNA levels and presented as fold increases compared to the controls. Below are the primer sequences utilized for the tested genes: *caspase-3* Forward 5'-AGAGGGGATCGTTGTAGAAGTC-3' and Reverse 5'-ACAGTCCAGTTCTGTACCACG-3'; *bcl2* Forward 5'-CCAGCGTATATCGGAATGTGG-3' and Reverse 5'-CCATGTGATACCTGCTGAGAAG-3'; *bax* Forward 5'-CCCGAGAGGTCTTTTTCCGAG-3' and Reverse 5'-CCAGCCCATGATGGTTCTGAT-3'; *β-actin* Forward 5'-TACCACTGGCATCGTGATGGACT-3' and Reverse 5'-TCCTTCTGCATCCTGTCGGCAAT-3'.

### 5.6 Histological Analysis

The central regions of the left lung tissue specimens were fixed in 4% paraformaldehyde, followed by embedding in paraffin and subsequent sectioning (5 μm). These sections underwent staining procedures, including Hematoxylin-Eosin (H&E) staining, TUNEL nuclear staining, and Immunohistochemistry (IHC). Myeloperoxidase is an important indicator of neutrophil activation and is associated with the absolute number of neutrophils. The IHC staining method utilizing the avidin-biotin complex was employed for neutrophil detection. After deparaffinization and blocking with 10% goat serum, the sections were incubated with a primary antibody (anti-myeloperoxidase antibody), followed by exposure to a biotin-labeled secondary antibody, following standard procedures. Cell apoptosis was detected using TUNEL staining. The assessment of perivascular edema progression involved a comparative analysis between the perivascular area and the entirety of the vessel area. All the scores were repeated three times using a double-blind method. TUNEL staining (*In situ* Death Cell Detection Kit, Roche, Belgium) with DAB staining was performed following the manufacturer’s instructions. The quantification of myeloperoxidase-positive cells and apoptotic cells involved determining the mean count within 5 randomly selected High-power Fields (HPFs) per section, observed at a magnification of 400×. The evaluation of the perivascular cuff area was conducted in 5 randomly chosen vessels per section, observed under a magnification of 200×.

### 5.7 The Ratio of Lung Wet Weight to Dry Weight

In order to calculate the lung wet-to-dry weight ratio, the upper section was utilized for calculations. Immediately following harvest, wet weights were recorded, while dry weights were measured subsequent to a 24-hour drying period at 100°C. This specific ratio was derived by dividing the wet weight with the dry weight.

### 5.8 Enzyme-linked Immunosorbent Assay (ELISA)

10 mg of lower left lung tissues underwent homogenization in 600 μl of PBS supplemented with protease inhibitors (Roche, Belgium). The tissue lysate was centrifuged at 12500rpm for 15 minutes, being the cleared supernatant collected and used for ELISA assay. Levels of TNF-α, IL-6, IL-8, and IL-10 in lung tissue lysates were quantified using ELISA kits in accordance with the manufacturer's instructions.

### 5.9 Statistical Analysis

The data are presented as mean ± standard error of the mean. To compare two independent groups, the Mann–Whitney U test was employed for the statistical analysis. GraphPad Prism Software (Version 8; GraphPad Software Inc, La Jolla, Calif) was also used for the statistical analysis. Adjusted *p*-values for multiple comparisons were acquired through the Tukey post-hoc test, with statistical significance determined at *p* < 0.05.

## 6 RESULTS

### 6.1 Natural Compounds Screening Identified Reb B to Exhibit a Positive Modulatory Effect on Cell Viability in the A549 Cell OGD/R Model

**Fig. (1):**
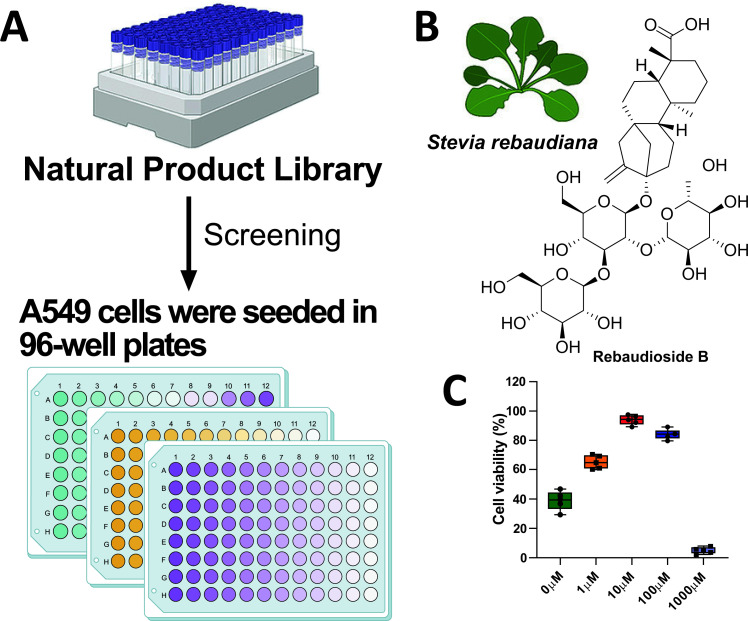
Reb B was identified through high-throughput screening of the OGD/R model of A549 cells. The diagram shows the screening approach (**A**). The chemical structure of Reb B is shown (**B**). Reb B, Rebaudioside B; OGD/R, Oxygen-glucose deprivation/recovery. Cell viability of various treatment concentrations of Reb B on OGD/R of A549 cells. In the OGD/R model of A549 cells, the cell viability was the highest when the Reb B exposure concentration was 10 mM, but it gradually decreased with the increase in Reb B concentration (**C**).

Fig. (**1A**) illustrates the schematic diagram of the research design; the protection was measured as presented in percentage, representing the cell viability assay. With this experiment was then selected the most promising candidate compound for the following experiments, namely Reb B (Fig. **1B**). The CCK8 assay was utilized to examine the impact of varying concentrations of Reb B on the viability of A549 cells under OGD/R conditions. The cell viability obtained was 42.71%, 68.13%, 96.14%, 85.30%, and 7.13% for 0 mM, 1 mM, 10 mM, 100 mM, and 1000 mM of Reb B, respectively (Fig. **1C**). Therefore, 10 mM of Reb B was used for further experiments.

### 6.2 GO enrichment Analysis

**Fig. (2):**
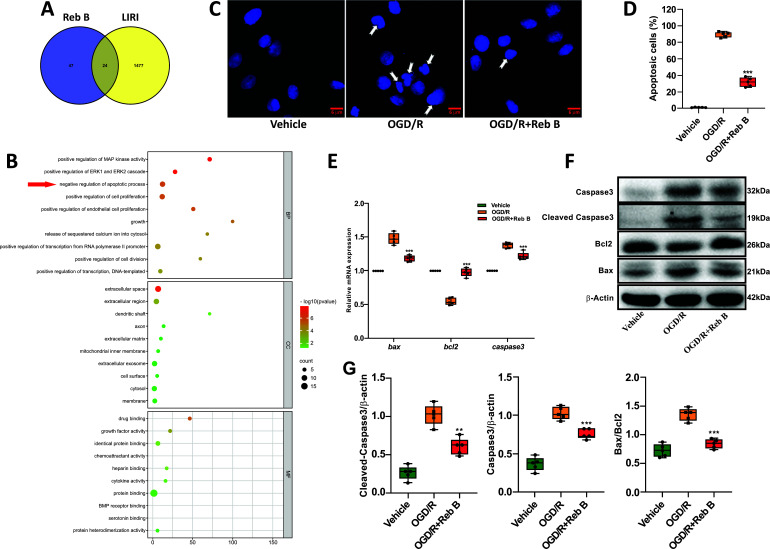
Venn diagram of Reb B drug targets and LIRI disease targets (**A**). GO enrichment analysis bubble chart. The top 10 enriched terms for each category, along with their corresponding *p*-values, are presented in a dot plot format. The horizontal axis quantifies the count of targeted genes, with the left segment delineating the classifications as BP (Biological Process), CC (Cellular Component), and MF (Molecular Function). Colors reflect the *p*-values, with smaller *p*-values leaning toward red and larger P-values toward blue (**B**). Analysis of apoptotic cells staining with Hoechst 33258 in the vehicle group, OGD/R group, and OGD/R+Reb B group (**C**) (original magnification: 400´). In comparison to the OGD/R group, there was a significant reduction in apoptotic cells observed in the OGD/R+Reb B group (**D**). Quantitative mRNA analysis of apoptosis-related genes (**E**). Representative western blot showing changes in the expression of proteins associated with apoptosis (**F**). Quantitative analysis of western blot analysis targeting apoptosis-related proteins (**G**). Arrows indicate representative apoptotic-positive cells. Data are expressed as mean ± standard deviation. **p < 0.01 *versus* OGD/R group, ***p < 0.001 *versus* OGD/R group. Reb B, Rebaudioside B; OGD/R, Oxygen-glucose deprivation/recovery.

The identified drug targets and disease-related targets were cross-referenced using Venny 2.1 software, resulting in 24 common targets (Fig. **2A**). Subsequently, we conducted a Gene Ontology (GO) enrichment analysis of these 24 shared targets using the DAVID 6.8 database to elucidate the potential roles of the candidate targets. The outcomes from the GO enrichment analysis (Fig. **2B**) unveiled that the key targets interacting with Reb B in the context of LIRI were primarily involved in positive regulation of MAP kinase activity, positive regulation of ERK1 and ERK2 cascade, negative regulation of apoptotic processes, positive regulation of cell proliferation, positive regulation of endothelial cell proliferation, and other biological processes. Additionally, these targets were associated with functions, such as drug binding, growth factor activity, protein binding, chemoattractant activity, and additional molecular functionalities. In terms of cellular components, they encompassed extracellular space, extracellular region, dendritic shaft, axon, and membrane, among others.

### 6.3 Effect of Reb B on Apoptosis of OGD/R in A549 Cells

Based on the outcomes of the GO enrichment analysis, it was evident that Reb B exerted a negative regulatory influence on the cellular apoptosis process within the context of lung ischemia-reperfusion injury. Consequently, further analysis of the effect of Reb B on cell apoptosis was conducted using the A549 cell OGD/R model. Hoechst33258 staining revealed the presence of many apoptotic cells after OGD/R treatment (Fig. **2C**). The OGD/R+Reb B group showed a significantly lower apoptosis rate compared to the OGD/R group (31.86 ± 5.64 *vs.* 89.58 ± 3.21; *p* <0.001, Fig. **2D**). In the OGD/R+Reb B group, RT-qPCR analysis revealed a notable decrease in the caspase-3/β-actin and bax/β-actin levels compared to the OGD/R group. However, the bcl2/β-actin level in the OGD/R+Reb B group was higher than that observed in the OGD/R group (Fig. **2E**). Similarly, WB analysis showed the caspase-3/b-actin, cleaved-caspase-3/b-actin, and Bax/Bcl_2_ levels in the OGD/R+Reb B group to be significantly reduced compared to the OGD/R group (0.76 ± 0.06 *vs.* 1.03 ± 0.08, *p* < 0.001; 0.61 ± 0.11 *vs.* 1.02 ± 0.13, *p* < 0.001, and 0.84 ± 0.08 *vs.* 1.35 ± 0.11, *p* < 0.001, respectively; (Figs. **2F** and **G**)).

### 6.4 Histological Effect of Reb B

**Fig. (3):**
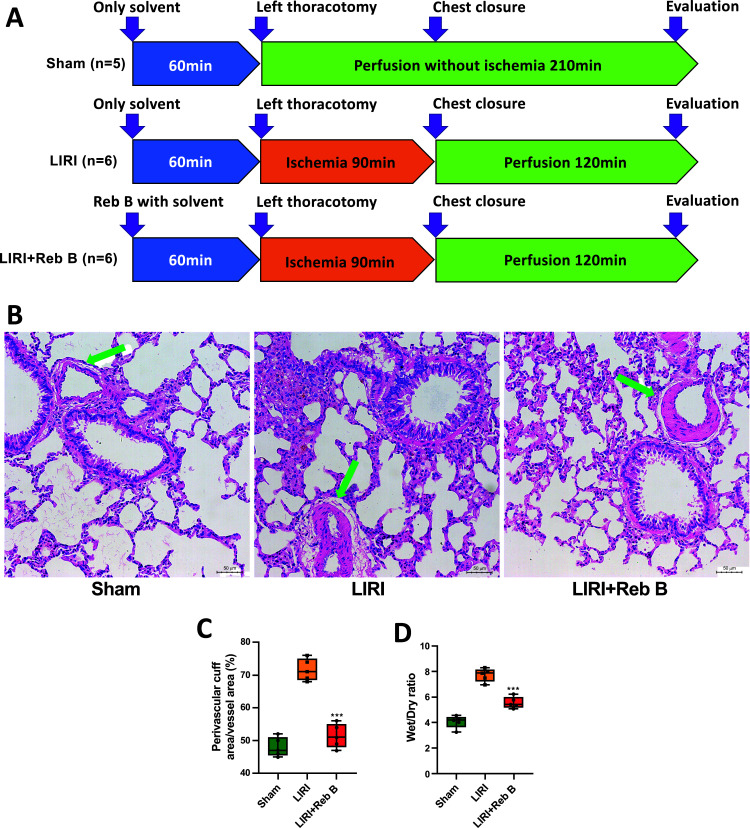
Illustration detailing the experimental protocol. Three experimental groups were conducted, with rats randomly allocated to the following: Sham group (n=5), LIRI group (n=6), and LIRI+Reb B group (n=6) (**A**). Histological sections illustrating lung samples from the Sham group, LIRI, and LIRI+Reb B group (**B**). The perivascular edema evaluated using a vascular cuff was also ameliorated in the LIRI+Reb B group (**C**). Arrows indicate representative perivascular edema. Wet-to-dry weight ratio (**D**). Data are represented as mean ± SD. ***p < 0.0001 *versus* LIRI group. Reb B, Rebaudioside B; LIRI, Lung ischemia-reperfusion injury.

In order to examine the *in vivo* impact of Reb B, we created a lung ischemia-reperfusion model in rats. The procedural details are elucidated in Fig. (**3A**). The assessment of the perivascular cuff area encompassed 5 vessels per histologic section, amounting to 25 vessels for the Sham group and 30 vessels for both the LIRI and LIRI+Reb B groups. The calculation of the perivascular cuff area index was carried out relative to the vessel area to mitigate variations associated with vessel size [8]. In the LIRI+Reb B group, the index was notably decreased compared to the LIRI group (51.40 ± 3.65 *vs.* 71.60 ± 3.36, *p* < 0.001; (Figs. **3B** and **C**)), while the wet-to-dry weight ratio similarly indicated a significant decrease in the LIRI+Reb B group compared to the LIRI group (5.57 ± 0.45 *vs.* 7.73 ± 0.51, *p* < 0.001; (Fig. **3D**)).

### 6.5 The Impact of Reb B on Apoptosis in Pulmonary Tissue and Proteins Associated with the Mitochondrial Apoptotic Pathway

**Fig. (4):**
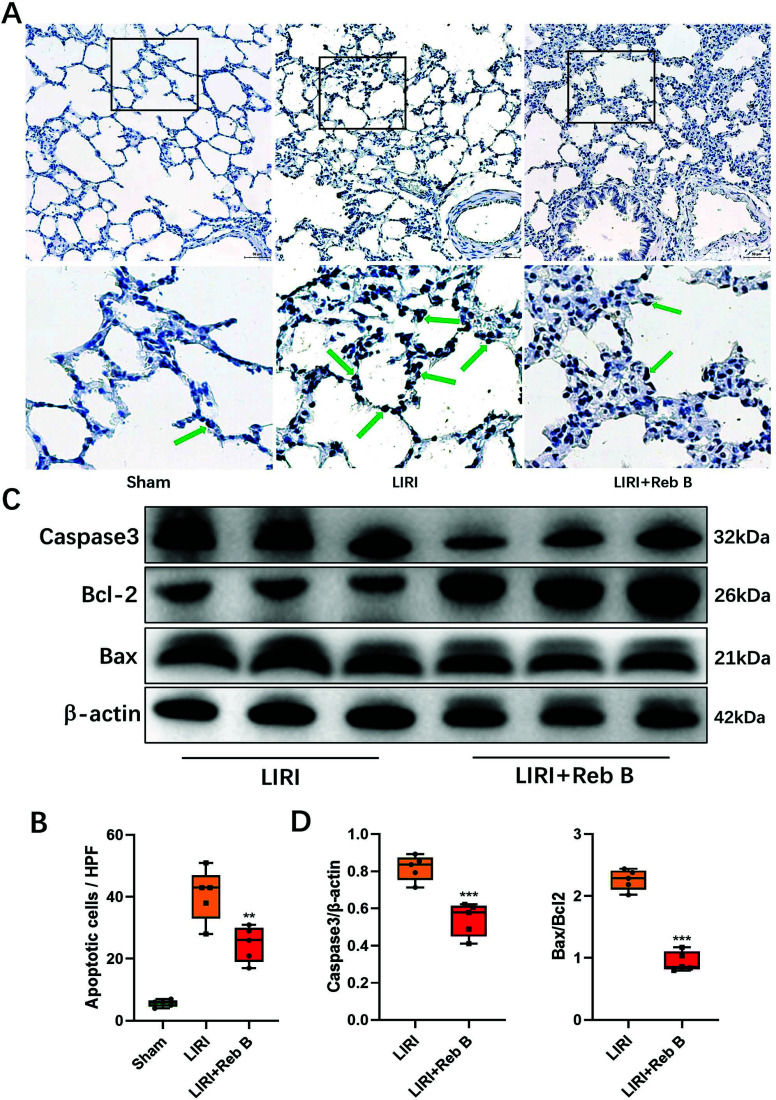
Representative images of TUNEL staining in the Sham group, LIRI group, and LIRI+Reb B group (**A**). The count of TUNEL-positive cells exhibited a significant decrease in the LIRI+Reb B group (**B**). Exemplary western blot depicting alterations in apoptosis-related protein expression within lung tissue (**C**). Quantitative analysis of western blot of apoptosis-related proteins (**D**). Arrows indicate representative apoptotic-positive cells. Data are reported as mean ± SD. **p < 0.01 *versus* LIRI group, ***p < 0.001 *versus* LIRI group. Reb B, Rebaudioside B; LIRI, Lung ischemia-reperfusion injury.

Based on TUNEL staining, there was a decrease in the quantity of apoptotic cells observed in the LIRI+Reb B group when compared to the LIRI group (24.80 ± 5.76 *vs.* 40.60 ± 8.44, *p* < 0.001; (Figs. **4A** and **B**)). The results obtained concerning the effects of Reb B on mitochondrial apoptosis were similar compared to the results of the *in vitro* cells. WB analysis showed that the caspase-3/b-actin and Bax/Bcl2 levels in the LIRI+Reb B group were significantly reduced compared to the LIRI group (0.54 ± 0.09 *vs.* 0.82 ± 0.07, *p* < 0.001, and 0.94 ± 0.16 *vs*. 2.26 ± 0.17, *p* < 0.001, respectively; (Figs. **4C** and **D**)).

### 6.6 The Effect of Reb B on Inflammation and Cytokine Levels

**Fig. (5):**
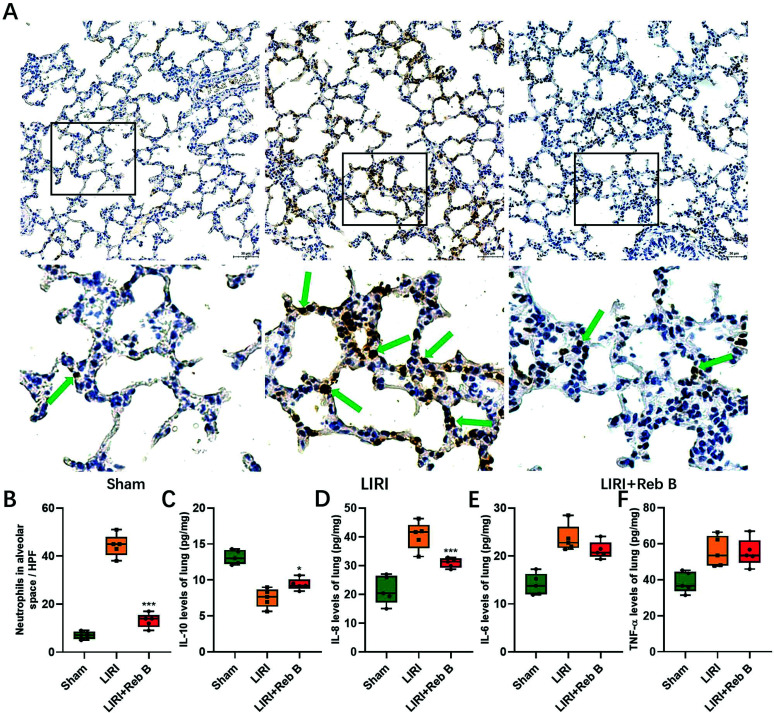
Representative IHC images for myeloperoxidase in the Sham group, LIRI group, and LIRI+Reb B group (**A**). The count of myeloperoxidase-positive cells showed a significant reduction in the LIRI+Reb B group (**B**). Level of TNF-a, IL-6, IL-8, and IL-10 in lung tissue lysates of the 3 groups: Sham group, LIRI group, and LIRI+Reb B group (**C**-**F**). Arrows indicate representative myeloperoxidase-positive cells. Data are represented as mean ± SD. *p* < 0.05 *versus* LIRI group, ***p < 0.001 *versus* LIRI group. IHC, Immunohistochemistry; Reb B, Rebaudioside B; LIRI, Lung ischemia-reperfusion injury.

In the LIRI+Reb B group, there was a significant reduction observed in neutrophil infiltration within the alveolar area compared to the LIRI group (13.20 ± 2.95 *vs.* 44.40 ± 4.67, *p* < 0.001; (Figs. **5A** and **B**)). IL-10 levels exhibited a significant increase in the LIRI+Reb B group compared to the LIRI group (9.39 ± 0.81 pg/mL *vs.* 7.51 ± 1.30 pg/mL, *p* < 0.05) (Fig. **5C**). In the LIRI+Reb B group, the levels of IL-8 demonstrated a significant reduction compared to the LIRI group (30.95 ± 1.68 pg/mL *vs.* 40.40 ± 4.84 pg/mL, *p* = 0.003) (Fig. **5D**). The levels of TNF-a and IL-6 were not significantly lower in comparison to the LIRI group (55.21 ± 7.65 pg/mL *vs.* 55.57 ± 8.43 pg/mL, *p* = 0.94, and 21.20 ± 1.791 pg/mL *vs.* 23.65 ± 2.823 pg/mL, *p* = 0.14, respectively; (Figs. **5E** and **F**)).

## 7 DISCUSSION

The results presented in the current study suggest that Reb B may exert a significant *in vitro* and *in vivo* lung protective effect. To our understanding, this study is the pioneering demonstration of Reb B's ability to inhibit apoptosis within lung tissues and its consequential therapeutic impact on lung injury in an *in vivo* LIRI model. Furthermore, Reb B has the potential to reduce the count of apoptotic cells and lower proinflammatory cytokine levels in lung tissues.

In this context, compounds from natural sources constitute promising candidates in pharmacotherapy to prevent and treat several diseases [9]. Natural compounds have advantages compared to conventional drugs, namely higher efficacy and lower toxicity, and for that reason, several natural compounds have been used in clinical practice [10]. The utilization of high-throughput screening technology has enabled the exploration of compound libraries to pinpoint lead compounds, which can subsequently undergo development into valuable therapeutic agents [11]. Before conducting animal experiments, human cell lines have been extensively employed in high-throughput drug screening processes [12]. Consequently, the widely utilized human alveolar epithelial cell line A549, known for its ability to simulate lung-related diseases *in vitro*, was employed in this study. Thus, a natural product library that consisted of 2,661 compounds was used, with Reb B being the most promising compound, exhibiting potent anti-apoptotic ability against the OGD/R model of A549 cells. Reb B constitutes a significant component of SGs, which are abundantly present in Stevia rebaudiana leaves [5]. SGs have gained recognition for their intense sweetness (250-300 times sweeter than sucrose), leading to their use as a non-caloric sweetener in numerous countries [13, 14]. Furthermore, SGs exhibit the ability to inhibit TNF-α and stimulate IL-8 release in intestinal cells. This effect is achieved through the inhibition of Nuclear Factor-kappa B (NF-κB) activation. Additionally, in Lipopolysaccharide (LPS)-stimulated THP-1 cells, SGs interfered with the IKKβ and NF-κB signaling pathway, thereby suppressing the production of inflammatory cytokines [13, 15, 16]. The findings of this study substantiate Reb B's capacity to exert an anti-apoptotic effect by impeding the mitochondrial apoptotic pathway. As a result, this pathway was further investigated in the subsequent *in vivo* study.

## CONCLUSION

Currently, there is no effective treatment for LIRI. Cell apoptosis generally engages two primary signaling pathways: the mitochondrial apoptotic pathway and the death receptor apoptotic pathway [17]. Members of the Bcl-2 family govern the mitochondrial pathway of apoptosis [17]. In LIRI, the levels of Bcl-2 are decreased, while the levels of cleaved caspase-3 are increased in the lung tissue to activate lung cell apoptosis. The outcomes of this investigation propose that administering Reb B enhances physiological functionality by inhibiting mitochondrial apoptosis pathways in injured lungs. Neutrophil infiltration and its resulting by products, such as peroxidases and proteinases, can severely damage the lung tissue [18].

LIRI injury prompts the infiltration of neutrophils into the alveoli, thereby triggering elevated production levels of inflammatory cytokines. The administration of Reb B before LIRI significantly reduces neutrophil infiltration. Regarding the levels of cytokines, the decreased level of IL-8 and increased IL-10 levels in the lung occurred in this study. The level of IL-8 produced correlates with the severity of lung injury [19]. IL-8, a proinflammatory cytokine, promotes chemotaxis and degranulation in neutrophils [20]. Furthermore, significant attenuation of lung injury induced by ischemia-reperfusion injury has been observed upon the blockade of IL-8 [21]. IL-10 is an anti-inflammatory cytokine that controls the inflammatory response [22]. Gene therapy using this cytokine has already been shown to be effective for the treatment of injured donor lungs [23]. Interestingly, IL-6 and TNF-a levels also decreased, but without statistical significance.

## LIMITATIONS

This study has several limitations. Despite the single administration of Reb B (5 mg/kg) preceding the ischemic event, notable enhancements were observed in the context of LIRI. Therefore, in forthcoming studies, acquiring more comprehensive data through experiments involving varied doses and administration frequencies of Reb B would be imperative. On the other hand, Reb B affects the entire organism and not only the lungs, and consequently, the effect of Reb B on the other organs should be further investigated. Male Sprague Dawley (SD) rats with a similar age were used in this study, but the potential influence of gender, age, hormone level, and other factors, on the results was not studied.

In conclusion, Reb B administration before ischemia attenuated LIRI *via* inhibition of apoptosis, proinflammatory cytokines production, and neutrophil infiltration in a rat model.

## Data Availability

The data and supportive information are available within the article.

## LIST OF ABBREVIATIONS

BCA: Bicinchoninic Acid Assay
CCK8: Cell Counting Kit-8
DMEM: Dulbecco's Modified Eagle Medium
DMSO: Dimethylsulfoxide
ELISA: Enzyme-linked Immunosorbent Assay
FBS: Fetal Calf Serum
H&E: Hematoxylin-eosin
HPFs: High-power Fields
IHC: Immunohistochemistry
LIRI: Lung Ischemia-reperfusion Injury
OGD/R: Oxygen-glucose Deprivation/Recovery
PVDF: Polyvinylidene Fluoride
Reb B: Rebaudioside B
RT-qPCR: Reverse Transcription-Quantitative PCR
SDS-PAGE: Sodium Dodecyl Sulfate-polyacrylamide Gel Electrophoresis
SGs: Steviol Glycosides
